# How to Measure Moral Realism

**DOI:** 10.1007/s13164-018-0401-8

**Published:** 2018-06-01

**Authors:** Thomas Pölzler

**Affiliations:** Institute of Philosophy, University of Graz, Graz, Austria

## Abstract

In recent years an increasing number of psychologists have begun to explore the prevalence, causes and effects of ordinary people’s intuitions about moral realism. Many of these studies have lacked in construct validity, i.e., they have failed to (fully or exclusively) measure moral realism. My aim in this paper accordingly is to motivate and guide methodological improvements. In analysis of prominent existing measures, I develop general recommendations for overcoming ten *prima facie* serious worries about research on folk moral realism. G1 and G2 require studies’ answer choices to be as metaethically comprehensive as methodologically feasible. G3 and G4 prevent fallacious inferences from intuitions about related debates. G5 and G6 limit first-order moral and epistemic influences. G7 address studies’ instructions. And G8 and G9 suggest tests of important psychological presuppositions.

## Introduction

Moral realism and anti-realism have been understood in various different ways (Boyd 1988; Dummett 1978; Sayre-McCord 1988). On what is probably the most common definition, they are about the existence of objective (i.e., observer-independent) moral truths (Brink 1989; Huemer 2005: 4; Joyce 2007a; Miller 2014; 2009). Realists believe that moral sentences are truth-apt, that some of these sentences are true, and that they are true in an objective sense. Anti-realists deny at least one of these claims. That is, according to them, moral sentences such as “Lying is morally wrong” or “We ought to maximize happiness” are either not truth-apt at all; are all false; or are, if true, only subjectively true.^1^

Discussions about moral realism have traditionally focused on the philosophical issue of its correctness (e.g., Ayer 1952; Brink 1989; Mackie 2011; Moore 1993). However, the existence of objective moral truths may also be addressed from a psychological perspective. One may study what, if anything, people *think* about this matter. Results of such investigations would not only enhance our understanding of (meta-)normative cognition; they may also have significant philosophical and practical implications. For example, it has been argued that as people experience morality as a realm of objective truths there is a presumption in favour of it being such a realm (e.g., Dancy 1986: 172; McNaughton 1988: 40); or that regarding an action’s wrongness as objective makes one more likely to prefer the criminalization of this action (Goodwin and Darley 2010: 181–184) and less tolerant towards those who deny the action’s wrongness (Wright et al. 2014).

Despite their significance, psychology has long neglected people’s intuitions about the existence of objective moral truths. Only in the last 15 years interest in the prevalence, causes and effects of these intuitions has increased. Sometimes under headings such as moral “objectivism”, “subjectivism”, and “relativism”, researchers have so far conducted around twenty studies about this issue,^2^ with a strong upward tendency (e.g., Beebe 2014, 2015; Beebe and Sackris 2016; Beebe et al. 2015; Cova and Ravat 2008; Fisher et al. 2017; Goodwin and Darley 2008, 2010, 2012; Heiphetz and Young 2017; Nichols 2004; Nichols and Folds-Bennett 2003; Sarkissian et al. 2011; Wainryb et al. 2004; Wright 2018; Wright et al. 2013, 2014; Young and Durwin 2013).^3^ These studies have proceeded by presenting scenarios that involve moral item statements, followed by questions with predefined answer choices. Most commonly, subjects were asked to interpret disagreements about moral statements or to assess these statements’ truth-aptness.

The outcomes of this first wave of studies on folk moral realism have been interpreted quite differently. At the beginning many researchers claimed that the studies support a tendency towards realism. Goodwin and Darley, for example, summed up the findings of their influential 2008 study as follows: “Individuals seem to identify a strong objective component to their core ethical beliefs […]. Arguably, many of our participants viewed their ethical beliefs as true in a mind-independent way” (2008: 1359; see also, e.g., Joyce 2006: 129–130). More recently, in contrast, people have been thought rather to favor realism with regard to some moral sentences and anti-realism with regard to others — depending on factors such as their openness to alternative moral views and their perceptions of consensus (e.g., Goodwin and Darley 2012; Pölzler 2017; Wright et al. 2013, 2014). The closer one looks, however, the more *any* such interpretation appears to be open to reasonable doubts.

One weakness of existing research on folk moral realism is that it has to some extent lacked in external validity. For example, the samples of the above-mentioned studies have so far often involved US students, i.e., highly educated Westerners between the ages of 19 and 24 (e.g., Goodwin and Darley 2008; Wright et al. 2013)^4^; the studies have typically only used item statements that involve “thin” (rather than “thick”) moral concepts, that are about actions (rather than persons or states of affairs), and that are about particular cases (rather than principles) (e.g., Beebe and Sackris 2016; Goodwin and Darley 2008; Nichols 2004)^5^; and their experimental stimuli have sometimes been inappropriately unrealistic and humorous (e.g., Sarkissian et al. 2011). I address these issues in more detail elsewhere (Pölzler 2018: 57-59; see also Bauman et al. 2014; Henrich et al. 2010).

In addition, and even more importantly, many studies on folk moral realism that have so far been conducted have also lacked in construct validity. Researchers have mostly started from appropriate definitions of moral realism and anti-realism, identical or similar to those introduced above; i.e., they have understood moral realism and anti-realism as the affirmation and denial of objective moral truths (see, e.g., the above quotation by Goodwin and Darley, and also Nichols 2004: 7; Nichols and Folds-Bennett 2003: B24; and fn. 3). But these views have then been improperly operationalized. Hence, the studies have failed to (fully or exclusively) measure those intuitions that subjects have about the existence of objective moral truths (see Beebe 2015; Moss 2017; Pölzler 2017).

That a study lacks in construct validity casts doubt upon this study. In order for psychology to move forward on the issue of folk moral realism more appropriate measures must hence be developed and employed as soon as possible. My aim in this paper is to motivate and guide such methodological improvements. In analysis of prominent existing measures, I will point out ten *prima facie* serious worries about psychological studies on folk moral realism. Some of these studies (W1) failed to cover variants of anti-realism, (W2) failed to cover variants of subjectivism, (W3) conflated the realism/anti-realism debate with the cognitivism/non-cognitivism debate, (W4) conflated the realism/anti-realism debate with the universalism/relativism debate, (W5) elicited strong first-order moral intuitions, (W6) elicited epistemic intuitions, (W7) involved problematic instructions, (W8) made unsubstantiated assumptions about subjects’ understanding of moral truth, or (W9) failed to account for the indirect relation between subjects’ responses and metaethical intuitions.

The extent to which these shortcomings are problematic varies. While some have decreased studies’ construct validity as a matter of conceptual necessity (W3 and W4), most of them only do so under empirical assumptions about subjects’ survey responses and intuitions. For example, ignoring certain variants of anti-realism (W1) and eliciting first-order moral (W5) or epistemic intuitions (W6) is only problematic if doing so notably and systematically influences response distributions. In my view, all of the above worries' underlying empirical assumptions are at least somewhat plausible; and some are highly plausible. But even if they ultimately turn out to be false — as long as this has not been shown psychologists clearly should not take the risk of ignoring these empirically contingent worries either. I will hence close my explanation of each of the above worries by developing recommendations for how to best alleviate them in future research.

## (G1) Account for the Main Variants of Anti-Realism

Metaethicists commonly distinguish three main ways of denying the existence of objective moral truths: non-cognitivism, error theory and subjectivism (e.g., Huemer 2005: 4–5; Joyce 2007a).

According to non-cognitivism, the reason for there not being objectively true moral sentences is that these sentences are not even truth-apt (at least not in a robust sense, see G7).^6^ In uttering such sentences people are rather thought to express non-cognitive mental states such as feelings of approval or disapproval, intentions or dispositions to have certain sentiments (see, e.g., Ayer 1952; Blackburn 2000; Gibbard 1990).

Error theorists are cognitivists, i.e., they believe that moral sentences are truth-apt. In contrast to realists, however, they hold that the facts that these sentences purport to refer to actually do not exist. This leads them to claim that all moral sentences are false — just as, for example, atheists believe that all theistic sentences are false, or most reasonable persons believe that all astrological sentences are false (see, e.g., Mackie 2011; Joyce 2001; Pigden 2007).^7^

Subjectivists, finally, hold both that moral sentences are truth-apt and that some of these sentences are true. Where they depart from realism is with regard to the question of what makes true moral sentences true. While realists believe that these sentences are made true by objective facts, subjectivists believe that the relevant facts are subjective, i.e., dependent on the mental states of observers (see, e.g., Firth 1952; Harman 1996; Hume 1978).

A first problem with many existing measures of moral realism is that their answer choices have failed to capture at least one of the above variants of anti-realism. Subjects who felt drawn towards these particular anti-realist variants were thus forced to respond in a way that did not reflect their actual intuition about the existence of objective moral truths. This may have led to these subjects being classified as favouring either an alternative variant of anti-realism (one that they actually did not favour) or even as holding realism (which they did not favour either).

Consider, for example, a recent measure by Beebe and Sackris (2016). In line with many other researchers Beebe and Sackris tried to determine subjects’ intuitions about the existence of objective moral truths by having them interpret cases of moral disagreement:

If someone disagrees with you about whether [Cutting the American flag into pieces and using it to clean one’s bathroom is morally wrong/Lying on behalf of a friend who is accused of murder is morally permissible/etc.], is it possible for both of you to be correct or must one of you be mistaken?

___ It is possible for both of you to be correct.

___ At least one of you must be mistaken. (Beebe and Sackris 2016: 922)

Beebe and Sackris took “It is possible for both of you to be correct” responses to be indicative of anti-realist intuitions, and “At least one of you must be mistaken” responses to be indicative of realist intuitions (2016: 922). However, in addition to other problems, this measure fails to account for the possibility of subjects being non-cognitivist anti-realists.^8^

Let us assume that subjects in Beebe and Sackris’ study understood the terms “correct” and “mistaken” in the correspondence-theoretic sense that is at issue in the moral realism/anti-realism debate (see G8). In this sense non-cognitivists deny *both* that it is possible for both parties of a moral disagreement to be correct *and* that at least one of them must be mistaken. On their account one cannot be correct or mistaken about moral sentences at all. Thus, subjects inclined towards non-cognitivism could not but choose an answer that conflicted with their actual intuition about whether the moral sentence they were presented with is objectively true. Prior to further empirical investigations we cannot rule out that this may sometimes have been the answer that Beebe and Sackris interpreted as indicative of realist intuitions (i.e., “At least one of you must be mistaken”).^9^

Given the possibly distorting effect of non-exhaustive answer choices, my first advice for researchers on folk moral realism is to provide answers which cover all of the three main variants of anti-realism. Beebe and Sackris’ measure, for example, could be improved along these lines by adding an option such as “Neither of you is correct or mistaken”. If subjects understood “correct” and “mistaken” in the sense that is at issue in the moral realism/anti-realism debate this option would exclusively appeal to non-cognitivists, and would hence prevent these subjects from responding according to moral realism.

## (G2) Account for the Main Variants of Subjectivism

Above I defined subjectivism as the view that moral sentences are made true by subjective moral facts. Metaethicists widely agree that for a moral fact to be subjective means that whether this fact obtains depends on the mental states of observers (see, e.g., Huemer 2005: 2–4; Miller 2014; Shafer-Landau 2003).^10^ But this conception is ambiguous. Most importantly, it fails to specify (1) in which sense moral properties must be independent from the mental states of observers, (2) from which observers’ mental states they must be independent, and (3) which particular mental states of these observers matter. Varying with one’s answers to these questions, subjectivism can be held in numerous different ways.

Individual subjectivists, for example, hold that an action is morally right, wrong, good, bad, etc. if and only if the individual who judges it in this way believes that it is right, wrong, good, bad, etc. Cultural relativists maintain that an action is morally right, wrong, good, bad, etc. if and only if the members of the culture in which the judgement is made predominantly believe that it has this property (see, e.g., Harman 1996).^11^ And according to response-dependence theorists, whether a moral sentence is true depends on how actual or hypothetical observers respond to that action under certain circumstances; for example, on whether humans tend to have certain emotions in response to the action (e.g., Hume 1978; Prinz 2006, 2007) or on whether an ideal observer would approve of it (e.g., Firth 1952).^12^

Besides disregarding variants of anti-realism as a whole, existing measures of moral realism have also often failed to provide answer options which reflect certain variants of subjectivism. In the case of highly exotic or highly sophisticated subjectivist views (e.g., Korsgaard 1996) this shortcoming may be of little consequence. Ordinary people’s intuitions presumably rarely favor such views. However, many studies have failed to cover variants of subjectivism that can reasonably be expected to be held by a more noteworthy proportion of the population as well (such as the variants presented above). Subjects who favored these variants thus had to respond in ways that did not reflect their actual intuitions about the existence of objective moral truths. This may have again led to the studies reporting wrong distributions of intuitions of this kind.

As an example, consider Wainryb et al.’s 2004 study on children between the age of five and nine. Subjects in this study were again presented a case of moral disagreement and asked to interpret this case:

Sarah believes that it’s okay to hit and kick other children, and Sophie believes that it’s wrong to hit and kick other children. […] Do you think that only one belief [what Sophie believes] is right, or do you think that both beliefs [what both Sophie and Sarah believe] are right? (Wainryb et al. 2004: 691)

Wainryb et al. interpreted “only one belief is right” responses as indicative of realist intuitions, and “both beliefs are right” responses as indicative of anti-realist intuitions (2004: 692). However, in addition to other problems, the answer that only one belief is right is not only entailed by realism, but also by many forms of non-individualistic subjectivism (Pölzler 2017: 464-465).^13^

As an example, consider cultural relativism. Proponents of this view believe that an action such as hitting and kicking other children is (morally) okay if and only if the members of the culture within which this judgement is made predominantly believe that the action is okay. Within one particular culture there can only be one predominant view about whether an action is okay. So as long as the parties of a moral disagreement are members of one and the same culture not only realists, but cultural relativists should be drawn to the option that only one of these parties is right as well.

With regard to Wainryb et al.’s study this observation is clearly relevant. By presenting subjects with drawings that show Sarah and Sophie standing face to face to each other (2004: 692) the researchers actively promoted the interpretation that these characters are members of one and the same culture. Hence, we do not know whether subjects who chose the “only one belief is right” option did so because they had intuitions in favor of realism (as assumed by Wainryb et al.) or whether they in fact favored cultural relativism.

A first step in improving Wainryb et al.’s above measure would be to ask subjects who chose the supposedly realist option to also interpret *cross-cultural* disagreement about whether it’s okay to hit and kick other children — say, between Faizah and Sophie rather than between Sarah and Sophie (see Nichols 2004; Sarkissian et al. 2011). To the extent that these subjects ceased to think that only one belief is right they would reveal themselves as cultural relativists rather than as realists (with regard to hitting and kicking other children). Subjects who also regarded one of the parties of cross-cultural moral disagreements as right, in contrast, would have to be asked further questions in order to determine their intuitions about the existence of objective moral truths. In particular, one would have to present them with tasks which allow to discriminate whether these subjects are realists or adhere to variants of non-individualistic subjectivism other than cultural relativism (such as response-dependence theory).

My above suggestions as to how to improve Wainryb et al.’s survey design show that testing for variants of subjectivism can require extensive and complex measures. But as has already been occasionally acknowledged (Sarkissian et al. 2011: 485–486), in terms of construct validity there is no way of escaping such intricacies. I hence advise researchers on folk moral realism to account more strongly for different variants of subjectivism; at least for those variants that can be expected to be held most widely.^14^

## (G3) Do not Conflate Moral Realism/Anti-Realism with Cognitivism/Non-Cognitivism

The moral realism/anti-realism issue is closely related to various other metaethical debates. One of these debates (which we already touched on above) concerns the truth-aptness of moral sentences, i.e., whether these sentences are either true or false or neither true nor false. Cognitivists believe that moral sentences are truth-apt. Non-cognitivists, in contrast, deny that these sentences can be assessed in terms of truth or falsity. They rather take them to be expressions of (non-truth-apt) desires.

Some studies on folk moral realism and anti-realism have inadvertently investigated subjects’ intuitions about the truth-aptness of moral sentences. As non-cognitivism is a variant of anti-realism, such results can allow conclusions about the minimum proportion of anti-realists in a given population. However, researchers have typically assumed that by investigating truth-aptness intuitions they illuminate folk moral realism and anti-realism in a comprehensive manner. This assumption is mistaken. For as follows from the definitions in G1, cognitivism does not entail realism.

To illustrate the above problem, consider one of the measures employed by Goodwin and Darley (2008).^15^ Subjects in this study were presented a moral sentence (such as “Anonymously donating a significant proportion of one’s income to charity is a morally good action” or “Consciously discriminating against someone on the basis of race is morally wrong”; 2008: 1361–1362) and then asked the following question:

How would you regard the previous statement? Circle the number. (1) True statement. (2) False statement. (3) An opinion or attitude. (Goodwin and Darley 2008: 1344)

As Goodwin and Darley interpreted subjects’ responses, both “true” and “false” responses indicate intuitions in favor of realism, and “opinion or attitude” responses intuitions in favor of subjectivism (see 2008: 1345). However, even if we abstract from other problems, by contrasting responses of these two kinds the study actually at best measured the prevalence of cognitivism versus non-cognitivism (Pölzler 2017: 461-463).

To begin with, considering a moral sentence to be “true” or “false” is not only consistent with realism, but also with all other variants of cognitivism, i.e., with subjectivism and error theory as well. Subjectivists believe that moral sentences are true or false depending on whether they correctly represent the subjective moral facts. Error theorists believe that all moral sentences are false. Moreover, the “opinion or attitude” option probably did not appeal to subjectivists either, but rather to non-cognitivists; for only the latter believe that moral sentences cannot be assessed in terms of truth or falsity at all (assuming a correspondence-theoretic sense of these notions, see G8).^16^

In sum, while cognitivism/non-cognitivism tasks may be part of more comprehensive measures of moral realism and anti-realism (indicating the minimal proportion of anti-realists among subjects), they cannot determine intuitions about these positions in full. My recommendation number three for measuring moral realism is hence not to ascribe such exaggerated relevance to purportedly cognitivist or non-cognitivist responses in the first place.

## (G4) Do not Conflate Moral Realism/Anti-Realism with Universalism/Relativism

Another important metaethical debate concerns the scope of moral sentences, i.e., how widely these sentences apply. Universalists believe that true moral sentences are true for any individual at any time and any place. Relativists, in contrast, relativize the truth of these sentences to particular individuals, times or places. For example, they may claim that while it is wrong for people in the twenty-first century to eat meat, it was not wrong for our early ancestors to do so.

Some researchers who set out to investigate folk moral realism and anti-realism actually (partly) measured subjects’ intuitions about universalism and relativism. This conflation is even more problematic than the one considered in the previous section. For while certain variants of realism and anti-realism have implications for the scope of moral sentences (for example, both individual and cultural relativism as introduced above imply that actions can be wrong for particular individuals or cultures but not for others), inferences in the reverse direction are invalid. Most importantly, neither does universalism entail the existence of objective moral truths nor does relativism entail their non-existence (see Joyce 2007b).

To better grasp this problem with universalism/relativism-based measures, consider Nichols and Folds-Bennett’s (2003) study on the prevalence of moral response-dependence theory among children. Subjects in this study were presented both moral sentences (e.g., “it is good for one monkey to help another hurt monkey”) and sentences about the instantiation of paradigmatically response-dependent non-moral properties (e.g., “grapes are yummy”) (2003: B26-B27). For each sentence that they agreed to subjects were then asked a question that was supposed to bring out the degree to which they consider this sentence universalizable.^17^

Now, think about a long time ago, before there were any people. There were still grapes [monkeys], just like the grapes [monkeys] now. Way back then, before there were people, were grapes yummy [was it good for one monkey to help another hurt monkey]? (Nichols and Folds-Bennett 2003: B27)

Nichols and Folds-Bennett assumed that only children who judge moral sentences to be as universal as paradigmatic response-dependence sentences have intuitions in favor of moral response-dependence theory (2003: B30-B31). But actually the question of the scope of moral sentences does not have any implications for the truth of response-dependence theory at all. In particular, neither does universalism entail non-response-dependence nor does relativism entail response-dependence.^18^

First, suppose the sentence “it is good for one monkey to help another hurt monkey” is temporally universal, i.e., true at all times. This does not entail that the sentence is made true by non-response-dependent facts. For example, its universal truth may just as well be grounded in the responses of an ideal observer. Suppose for an action to be good is for such an observer to respond to this action with approval (e.g., Firth 1952), and an ideal observer would respond to one monkey helping another hurt monkey with approval at all times. Then for one monkey helping another hurt monkey would be good at all times, both today and “a long time ago, before there were any people” — even though the actions’ goodness would be response-dependent.

Conversely, that the sentence “it is good for one monkey to help another hurt monkey” is only true at particular times does not entail that it is made true by response-dependent facts either. First, the action’s goodness/non-goodness may be explained by non-response-dependent subjective facts, i.e., by facts that depend on mental states other than reactive attitudes, such as on beliefs or intentions.^19^ Suppose, for example, for an action to be good requires that it is dominantly believed to be good within a human culture. Then one might claim that it was not good for one monkey to help another hurt monkey “before there were any people”, but it is good today, because today in contrast to then there are human cultures which dominantly believe that it is good for one monkey to help another hurt monkey. Second, it may even be an *objective* moral fact that for one monkey to help another hurt monkey is good today but was not good before there were people.

Given that universalism and relativism lack any implications for questions about the existence of objective moral truths, my fourth recommendation for improving the methods of research on folk moral realism is to simply avoid testing these positions in the context of this research.^20^ Measures such as Nichols and Folds-Bennett’s cannot provide any valid data about people’s intuitions about moral objectivity at all.^21^

## (G5) Avoid Prompting First-Order Moral Intuitions

Moral realism and anti-realism belong to metaethics. This means that rather than being or entailing judgements about what is actually morally right, wrong, good, bad, etc., they are philosophical views *about* such first-order moral judgements (e.g., Huemer 2005: 1–2).^22^ For example, affirming that there are objectively true moral judgements does not commit one to judging that eating meat is permissible, or that eating meat is impermissible; and denying that there are objectively true moral judgements does not commit one to making (or not making) any of these judgements either.

Another problem with many studies on folk moral realism is that they have not sufficiently accounted for moral realism and anti-realism’s moral neutrality. In particular, researchers have attempted to measure subjects’ intuitions about these views by using scenarios, questions and answer choices that may also have prompted strong first-order moral intuitions. Subjects’ responses in these studies may consequently partly be explained by these intuitions, rather than by their views about moral realism and anti-realism alone.^23^

As an example, consider an early study on folk moral realism by Nichols (2004). In this study Nichols describes how two members of different cultures, John and Fred, disagree about moral issues such as whether “[i]t’s okay to hit people just because you feel like it.” He then asks subjects how they interpret this disagreement:

It is okay to hit people just because you feel like it, so John is right and Fred is wrong.

It is not okay to hit people just because you feel like it, so Fred is right and John is wrong.

There is no fact of the matter about unqualified claims like “It’s okay to hit people just because you feel like it.” Different cultures believe different things, and it is not abso-lutely true or false that it’s okay to hit people just because you feel like it. (Nichols 2004: 9-10)

On Nichols’ interpretation, the first and second of the above options indicate intuitions in favor of moral realism, while the third reflects anti-realism (see 2004: 10). This measure is problematic (among others) because it did not sufficiently account for moral realism and anti-realism’s moral neutrality.

Consider Nichols’ first and second answer options: “It is okay to hit people just because you feel like it” and “It is not okay to hit people just because you feel like it”. Both of these options express first-order moral judgments. They reflect a positive (first sentence) or negative (second sentence) moral stance towards hitting other people just because you feel like it. This may have led subjects to interpret Nichols’ task as being about their first-order moral intuitions. More specifically, those who picked options one or two may have (partly) done so because they had the intuition that it is or is not okay to hit people just because you feel like it, and not (only or mainly) because they experienced this issue as objective (Beebe 2015: 17).

Avoiding first-order moral intuitions in studies on folk moral realism altogether may be methodologically infeasible.^24^ However, the influence of such intuitions can certainly be limited. In order to increase the validity of Nichols’ above measure, for example, one could merge the first two answers into one disjunction and formulate them exclusively in terms of rightness and wrongness. The resulting statement (“Either John is right and Fred is wrong, or Fred is right and John is wrong”) would appeal to subjects more independently of how strongly they believe that it is or is not okay to hit people just because you feel like it.^25^ In any case, our above considerations suggest that researchers on folk moral realism should try to avoid prompting first-order moral intuitions as far as doing so is methodologically feasible.

## (G6) Avoid Prompting Epistemic Intuitions

Metaethicists do not only address semantic issues (such as the cognitivism/non-cognitivism debate) and metaphysical ones (such as the moral realism/anti-realism debate). They also consider whether and how persons *know*, have *good reasons to believe* or are *certain* that (particular) actions are morally right, wrong, good, bad, etc.

Some existing measures of moral realism have prompted subjects to think about moral knowledge, justification or certainty. As there are some logical relations between these epistemic issues and the existence of objective moral truths, such measures do not need to be rejected right away.^26^ However, researchers would have to account for the fact that the above epistemic issues typically do not track the moral realism/anti-realism distinction straightforwardly. Most importantly, even if we do not know or do not have good reason to believe or are uncertain that an action is right, wrong, good, bad, etc. it might still be the case that the action is objectively right, wrong, good, bad, etc. And even if we do know or do have good reason to believe or are certain that an action is right, wrong, good, bad, etc. it might still be the case that the action only has these properties in a subjective sense.

Researchers on folk moral realism have not always displayed full awareness of the complex relationship between moral epistemology and metaphysics. As an example, consider another part of Goodwin and Darley’s above-mentioned 2008 study. In this part subjects were told that some other subject of their study had denied a moral sentence that they themselves had agreed to. Then they were asked to interpret this case of moral disagreement in one of the following four ways:

The other person is surely mistaken.

It is possible that neither you nor the other person is mistaken.

It could be that you are mistaken, and the other person is correct.

Other (Goodwin and Darley 2008: 1344).

Goodwin and Darley took “The other person is surely mistaken” responses to indicate realism, and both “It is possible that neither you nor the other person is mistaken” and “It could be that you are mistaken, and the other person is correct” to indicate subjectivism (2008: 1344–1345).

One problem with this measure is that it again prompts first-order moral intuitions. Moreover, responses in favour of the first and third of Goodwin and Darley’s answer options may also reflect epistemic intuitions (Beebe 2015: 15). In particular, some of the subjects who declared that “The other person is *surely* mistaken” may have done so because they were *certain* that their first-order moral judgement is correct. And some of the subjects who declared that “It *could be* that you are mistaken, and the other person is correct” may have done so because they were *uncertain* about it. They may have thought that given their own state of knowledge — not given the nature of moral sentences — it is possible that they are mistaken.

Like with most other worries raised here, the extent to and way in which these formulations affected the distribution of subjects’ responses can only be determined empirically. But some systematic influence along the above lines does seem plausible. As claims about moral certainty neither entail moral realism nor moral anti-realism, this influence would have to be regarded as distorting Goodwin and Darley’s measurement of intuitions about moral objectivity.

Similar and related to the case of first-order moral intuitions, epistemic influences on subjects’ responses can often be reduced or eliminated. Goodwin and Darley’s measure, for example, could be improved simply by omitting the term “surely” in option one and by substituting “It could be that you are mistaken” by “It is possible that you are mistaken” (or even better, as possibility too can be read in an epistemic sense and need not be appealed to here at all: “You *are* mistaken”; or still better, limiting also the influence of first-order moral intuitions: “*Either* you *or* the other person is mistaken”). My sixth recommendation for researchers on folk moral realism hence is to either use epistemic issues properly or else to formulate their scenarios, questions and answer choices in ways which do not promote epistemic interpretations at all.

## (G7) Be Careful with Instructions

Above I suggested that not even the most careful formulation of scenarios, questions and answer choices may fully prevent improper first-order moral or epistemic influences on subjects’ responses. One way of addressing this problem is to implement validity checks as they will be discussed in G9. In addition, researchers on folk moral realism may also attempt to limit such influences by providing subjects with instructions about the nature of their questions or the moral realism/anti-realism debate.

There are two forms that substantive instructions can take: positive (explaining what a study’s questions or the moral realism/anti-realism debate are about) and negative (explaining what they are not about). Negative explanations clearly have potential for increasing a study’s validity. For example, researchers may prevent common misunderstandings among subjects by explaining that their questions are not about the extent to which one *knows*, is *justified in believing*, or is *certain* about moral sentences (see Wright 2018: 11),^27^ or by explaining that these questions are distinct from the extent to which the general public or subjects themselves *agree* with the sentences (see G9, and in particular fn. 31). Positive instructions, in comparison, tend to be more risky — in particular, if they are not (only) about a study’s questions, but (also) about the metaethical debates that these questions are supposed to reflect.

For some purposes (such as for supporting the presumptive argument for moral realism mentioned in the introduction) studies on folk moral realism must yield data about subjects’ pre-theoretical intuitions, i.e., about responses that are immediate in the sense that they have not been consciously inferred from metaethical theories. Explanations about the nature of (variants of) moral realism and anti-realism may prevent this kind of immediacy. They may lead subjects to consciously adopt one such variant, and then to answer on the basis of this theory even if their answers do not match their more immediate intuitions.

Positive instructions may also be problematic to the extent to which they involve complex philosophical concepts such as “truth” or “objectivity”. Even if these concepts are explained (for example, as the “representation of moral facts” or as “independence of the mental states of observers”), subjects may still understand them in unintended ways (see G8, and fn. 27). In fact, this lack of control over ordinary people’s understanding of relevant philosophical concepts is presumably why researchers explore intuitions about moral realism by asking questions about more mundane issues (such as the interpretation of moral disagreement) in the first place.

Finally, it is difficult to formulate positive instructions in ways that do not bias or mislead subjects. Consider, for example, Wright’s recent study (2018) on the prevalence of moral cognitivism versus non-cognitivism.^28^ Prior to completing this study subjects were asked to read a one-page explanation of what it means for a sentence to be truth-apt and not truth-apt. Wright’s explanation of not truth-apt sentences included the following passage:

Consider […] claims like “Peanut butter ice cream is delicious” or “Jazz music is the best form of music ever invented” or “Riding on the roller coaster at Eliches is awesome!” […] these statements aren’t truth-apt. […] Some people enjoy the taste of peanut butter ice cream, others don’t; Some people have a great time riding the roller coaster at Eliches, others don’t. So, if one person said “Riding roller coasters is awesome!” and another person said “Riding roller coasters is absolutely terrifying!” it wouldn’t make sense to say that one of the two was correct and the other mistaken. This is because neither of these statements are intended to accurately reflect some fact about roller coaster riding – rather, they are expressions of people’s liking/disliking of or approval/disapproval for something (in this case, riding roller coasters). (Wright 2018: 12)

This explanation likely distorted the results of Wright’s study. In particular, it may have biased subjects towards non-cognitivism, i.e., the view that moral sentences are not truth-apt.^29^

One problematic aspect of Wright’s explanation is the examples that she provides for not truth-apt sentences: “Peanut butter ice cream is delicious”, “Jazz music is the best form of music ever invented”, and “Riding on the roller coaster at Eliches is awesome!” All of these sentences are practically normative, i.e., about the goodness of actions (rather than about the goodness of beliefs or about descriptive facts). This may have led subjects to mistakenly regard non-truth-aptness as a distinctive feature of practically normative sentences, which in turn may have inclined them towards considering moral sentences to be not truth-apt.

Moreover, the above examples also create the impression that more sentences admit of uncontroversial "not truth-apt" interpretations than actually do. Many philosophers believe that deliciousness and aesthetic goodness are constituted by human or ideal observers having certain kinds of pleasant experiences (e.g., Hume 1903; Zangwill 2001).^30^ Based on such accounts “Peanut butter ice cream is delicious” and “Jazz music is the best form of music ever invented” turn out truth-apt (true if relevant observers respond to peanut butter ice cream and jazz music by having certain kinds of pleasant experiences; false if they do not respond in such a way).

Finally, in explaining that sentences about the deliciousness of peanut butter ice cream and the awesomeness of riding a roller coaster are not truth-apt Wright notes that “[s]ome people enjoy the taste of peanut butter ice cream, others don’t” and that “[s]ome people have a great time riding the roller coaster at Eliches, others don’t”. These statements create the impression that widespread disagreement about a sentence either entails or suggests that the sentence is not truth-apt. However, no such relation holds. Even if people very widely disagree about a sentence it is still often either true or false (think of sentences about the existence of God or the murder of John F. Kennedy). Thus, to the extent that Wright’s explanation led subjects to regard a moral sentence as not truth-apt because they perceived this sentence to be subject to disagreement its effect was distorting as well.

Based on the above observations, my seventh recommendation for researchers on folk moral realism is to be careful with substantive instructions. While negative instructions may be helpful, positive ones should only be considered when a study does not purport to measure pre-theoretical intuitions, when the instructions properly explain relevant philosophical concepts (such as “moral truth” or “objectivity”), and when they do not mislead or bias subjects. The effect of these instructions should also be tested by means of validity checks, comprehension checks, pilot studies or post-study interviews.^31^

## (G8) Test Subjects’ Understanding of Moral Truth (Correctness, Rightness, etc.)

Most existing measures of moral realism are based on the notion of moral truth, or on notions that researchers have understood in terms of moral truth, such as being morally correct or right (i.e., making a true moral judgement). For example, we saw that subjects were asked whether they regard moral sentences as “true”, “false” or an “opinion or attitude” (Goodwin and Darley 2008) or whether they believe that in cases of moral disagreement only one person can be right (Beebe and Sackris 2016; Goodwin and Darley 2008; Nichols 2004; Wainryb et al. 2004).

This focus on moral truth is warranted. After all, as they are understood here, moral realism and anti-realism are essentially about moral truth. At the same time it is important to note, however, that realists and anti-realists disagree about the existence of objective moral truths in a very specific sense of “moral truth”. They affirm or deny these truths in a *correspondence-theoretic* sense, according to which for a moral sentence to be true is for it to represent a moral fact (Huemer 2005: 38–44; Sayre-McCord 2015). This means that in order for truth-based measures of moral realism to be valid subjects would have to understand moral truth (correctness, rightness, etc.) in this correspondence-theoretic sense as well.

But do subjects understand moral truth in this way? One general worry about truth-based measures of moral realism is that this assumption has not yet been tested. It is therefore possible that when a subject judges that a particular moral sentence is, say, “true” — as opposed to an “opinion or attitude” — she does not mean that the sentence represents a moral fact at all. Rather, she might only mean to reaffirm this sentence (deflationist theory of moral truth, see Blackburn 2000: 79; Gibbard 2003: x) or to express that the sentence can be part of a coherent system of sentences (coherentist theory of moral truth, see Dorsey 2006). On any of these latter interpretations subjects’ responses do not directly bear on the moral realism/anti-realism distinction.

Besides most other measures that were considered so far, the above problem also afflicts a third part of Goodwin and Darley’s 2008 study. In this part subjects were presented a number of moral sentences and then asked for each of these sentences whether they believe that there is a correct answer as to whether the sentence is true:

According to you, can there be a correct answer as to whether this statement is true?

[yes]

[no] (Goodwin and Darley 2008: 1351)

On Goodwin and Darley’s interpretation, “yes” responses indicate realist intuitions, while “no” responses indicate subjectivism (2008: 1352).

Our above considerations suggest that the question of whether there can be a correct answer as to whether a moral sentence is true at best allows distinguishing cognitivist intuitions (“yes”) from non-cognitivist ones (“no”) (see G3).^32^ But even this revised interpretation of Goodwin and Darley’s results would only hold if their subjects understood moral truth in a correspondence-theoretic sense, i.e., if they considered whether there can be a correct answer as to whether the moral sentence at issue represents a moral fact. And Goodwin and Darley do not provide any evidence in favor of this psychological assumption (Sinclair 2012: 168).

One way in which researchers may attempt to alleviate the above problem is by instructing subjects about the relevant notion of truth. This strategy was already addressed above (see G7).^33^ In addition, there are also ways in which people’s understanding of moral truth might be investigated as part of such studies or in independent studies. For example, researchers on folk moral realism may directly ask subjects to rate the plausibility of (intelligibly explained) conceptions of moral truth or they may present them with hypothetical scenarios in which a moral sentence represents a robust moral fact, is reaffirmed, is part of a coherent system, etc. and then ask them whether they would say that the sentence is true.^34^

It is not immediately obvious how subjects’ conception of moral truth is most validly tested. Maybe there is no good (quantitative) method of doing so at all. However, in order for the psychological study of folk moral realism to move forward the challenge of determining subjects’ understanding of moral truth (correctness, rightness, etc.) cannot be avoided. My eighth recommendation for improving the study of folk moral realism hence is to accompany truth-based measures with tests of subjects’ understanding of this notion or to test this understanding in independent research.

## (G9) Use Validity Checks

Suppose a study on folk moral realism complies with all of the guidelines that have been suggested so far. This would still fail to guarantee the study’s construct validity. One trivial reason for this fact is that in addition to the problems addressed in this paper, measures of moral realism can be flawed in numerous other ways as well. But there is also a more fundamental and serious general worry about research of this kind.^35^

Researchers have explored subjects’ intuitions about the existence of objective moral truths by asking them questions such as whether a moral statement is “true”, “false” or “an opinion or attitude” (Goodwin and Darley 2008: 1344) or whether in a case of moral disagreement “only one belief is right” or “both beliefs are right” (Wainryb et al. 2004: 691). We have seen that so far these measures’ answer choices have not fully and exclusively logically entailed variants of moral realism or anti-realism. But suppose they did. Even then the fact that a subject chooses a particular answer would fall short of establishing that s/he has an intuition in favor of the entailed variant. The subject’s response could be explained in at least three plausible alternative ways as well.

A first alternative explanation is that the subject does not have any intuition about the particular question that s/he is presented with. For example, it may neither strike him/her that the statement “Anonymously donating a significant proportion of one’s income to charity is a morally good action” is “true” nor that this statement is “false” or an “opinion or attitude”; or it might neither strike him/her as true that in a case of moral disagreement “only one belief is right” nor that “both beliefs are right” (see Bengson 2013: 13). This explanation may not seem particularly plausible. After all, studies’ scenarios, questions and answer choices typically appear to be sufficiently simple and clear to elicit intuitions. But subjects’ prompted study responses are open to two other alternative explanations as well.

Suppose a subject does have an intuition about a particular question. It then still cannot be taken for granted that his/her answer accurately reflects this intuition (for example, that a subject who chose “true” really has the intuition that the statement at issue is true, or that a subject who chose “only one belief is right” really has the intuition that only one party of a moral disagreement has a right belief) (see Bengson 2013: 15; Moss 2017: 186-188). Aberrant responses can have various reasons. Some of them are random (such as temporary inattentiveness or confusion) and may hence disappear at the level of mean values. However, subjects may also fail to respond in accordance with their intuitions due to systematic distortions. There is evidence, for example, that questions in studies on folk moral realism may be conflated with logically distinct questions about public consensus and personal agreement (Goodwin and Darley 2008: 1350, 1354, 2012: 253–254; Wright et al. 2013: 349).^36^

Finally, even if a subject has an intuition about a question and her answer accurately reflects this intuition this still does not warrant the conclusion that the variant of moral realism or anti-realism that is entailed by this answer appears true to the subject. This is mainly because the subject may lack any determinate intuition about the issue of moral objectivity at all. Claims about the correspondence-theoretic truth(−aptness) of moral judgments, the observer-independence of moral facts, and so on might just be too abstract and complex for the folk to have such intuitions. Neil Sinclair, for example, writes:

What defines realism is the view that moral judgments have a characteristic linguistic function, express states of mind with a characteristic representational function and (therefore) that their truth consists in correspondence between the representational content of such states and the moral way of the world. […] Ask the woman on the Clapham omnibus whether recreational torture is wrong and she may well reply that it is. Ask whether her judgment that torture is wrong is representative of moral reality, or made true by correspondence between the representational content of the mental state her judgment expresses and the distinctively moral state of the world and one is more likely to be faced with an uncomprehending silence. (Sinclair 2012: 168; see also Moss 2017: 189-191)

Given the above alternative explanations, how can researchers make sure that subjects’ prompted answers reflect intuitions in favor of the entailed variants of moral realism and anti-realism? One possible way of alleviating this problem is again to provide instructions about the studies’ subject matter (see G7). First and foremost, however, researchers need to implement validity checks. Some of these checks may be quantitative. For example, to test whether subjects have determinate intuitions about studies’ questions and about the existence of objective moral truths at all, measures of confidence and response time may be helpful (the more confident subjects declare to be and the faster they respond the more likely they are to have such intuitions). In the end, the methodology of research on folk moral realism should probably be expanded to include open-ended questions, post-survey interviews, and other qualitative elements as well (see Moss 2017). Following Wright et al. (2013: 349–352), for example, one may ask subjects to provide verbal explanations of their responses, and then only include them in analysis if these explanations roughly match the variants of moral realism and anti-realism that are entailed by their responses.

The problem that subjects’ answers may not reflect intuitions in favor of entailed variants of moral realism and anti-realism is fundamental and serious. Considering also some of the other problems addressed here, one may begin to wonder whether (quantitative) scientific methods are appropriate for testing folk intuitions about moral realism at all (or, for that matter, about any philosophical claim; see, e.g., Kauppinen 2007). In any case, our above considerations have shown that if one does bring such methods to bear on the issue then validity checks are necessary to maximize one’s prospects of success. To include such checks is hence my last recommendation for researchers on folk moral realism.

## Conclusion

In recent years an increasing number of psychologists have begun to explore the prevalence, causes and effects of ordinary people’s intuitions about moral realism. Many of these studies lack in construct validity, i.e., they have failed to (fully or exclusively) measure moral realism. In this paper I therefore developed a manual for studies of this kind. In particular, I argued that in investigating folk moral realism psychologists ought to comply as much as possible with the following nine guidelines:

1. Account for the Main Variants of Anti-Realism.
2. Account for the Main Variants of Subjectivism.
3. Do not Conflate Moral Realism/Anti-Realism with Cognitivism/Non-Cognitivism.
4. Do not Conflate Moral Realism/Anti-Realism with Universalism/Relativism.
5. Avoid Prompting First-Order Moral Intuitions.
6. Avoid Prompting Epistemic Intuitions.
7. Be Careful with Instructions.
8. Test Subjects’ Understanding of Moral Truth (Correctness, Rightness, etc.).
9. Use Validity Checks.

Complying with these guidelines will not guarantee the success of any study on folk moral realism. First, for a study to be fully valid it must have external in addition to construct validity as well. Second, some of the problems that I have identified (such as the possibility that ordinary people lack any determinate intuitions about the truth of moral realism) may turn out not to be sufficiently preventable by experimental methods. And third, measures of moral realism can be flawed in many ways other than those addressed by the above recommendations too. Despite some recent improvements (e.g., Goodwin and Darley 2012; Sarkissian et al. 2011; Wright 2018), there is thus still a long way to go in achieving a more solid understanding of ordinary people’s thinking about the existence of objective moral truths.

Let me close by sketching what a first step in approaching such an understanding might look like in practice. Our above considerations suggest that one promising way of measuring ordinary people’s intuitions about the existence of objective moral truths is disagreement-based. A more valid measure of this kind may begin with asking subjects to interpret cases of disagreement about a number of item statements:

Two persons disagree about whether [action/person/states of affairs] is [morally good, bad, etc.]. One person judges that [action] is [morally good, bad, etc.]. The other person judges that it is not the case that [action/person/states of affairs] is [morally good, bad, etc.]. Which of the following interpretations of this disagreement best fits your view?

1. One of the persons is right and the other one is wrong. [potentially indicates realism or non-individual subjectivism]
2. Both persons are right. [potentially indicates individual subjectivism]
3. Both persons are wrong. [potentially indicates error theory]
4. Neither person is right or wrong. [potentially indicates non-cognitivism]

The above answer options are as comprehensive as possible in their coverage of moral anti-realism (G1) and do not conflate moral realism or anti-realism with other metaethical positions (G3, G4). They also prompt as little first-order and epistemic intuitions as methodologically feasible (G5, G6). Yet, in order for this measure to be valid it must also be supplemented by at least three additional tasks.

First, the study must test subjects’ understanding of what it means for a person to be “right” or “wrong” about a moral sentence (G8). Second, subjects must explain (in their own words) why they interpreted the disagreement according to the answer option that they chose (G9). And third, if a subject chooses “One of the persons is right and the other one is wrong” she must be asked to interpret similar cases of disagreement that allow discerning whether she is a realist or favors non-individualistic variants of subjectivism (for example, a task that presents the disagreeing parties as members of different cultures, with each of the parties’ moral judgements conforming to the majority view of their culture; G2).

A subject’s response to the above disagreement task only counts as reflecting her intuitions about the existence of objective moral truths if the subject understands “right” and “wrong” in terms of the correct representation of moral facts and the explanation of her response roughly corresponds to the metaethical position that is entailed by this response. Suppose both of these conditions are met. Then “One of the persons is right and the other one is wrong” answers reflect intuitions in favor of realism or non-individualistic subjectivism (depending on subjects’ responses to the third of the above-mentioned follow-up tasks), “Both persons are right” answers reflect intuitions in favor of individual subjectivism, “Both persons are wrong” answers reflect intuitions in favor of error theory, and “There is no such thing as being right or wrong” answers reflect intuitions in favor of non-cognitivism.

Needless to say, this design will have to be tested and refined in a series of actual experiments. From the theoretical perspective developed in this paper, however, it provides a promising starting point for approaching a more valid measurement of folk moral realism.

## Footnotes

1 This definition has also been assumed by most researchers on folk moral realism (see below). Yet it is important to note that the overwhelming majority of the guidelines developed in this paper hold on most plausible alternative definitions of moral realism and anti-realism as well (at least following straightforward adjustments). Thus, even if one believes that the moral realism/anti-realism debate concerns whether moral sentences are either determinately true or determinately false (Dummett 1978: 146), or if one prefers a different conception of objectivity than the one assumed here (e.g., Horgan and Timmons 2008: 270), one should be able to find much of interest.

2 This is just a rough estimate. As it can be unclear whether a psychological study is relevant to the issue of folk moral realism, the number of these studies may reasonably be considered lower or higher than suggested as well.

3 Some metaethicists (e.g., Björnsson 2012: 369) use the label “objectivism” to refer to positions that are distinct from moral realism as it was introduced here. The above mentioned researchers on folk metaethics, in contrast, mostly do refer to the existence of objective moral truths. According to Goodwin and Darley, for example, objectivists claim that moral beliefs or standards “derive their truth (or warrant) independently of human minds (i.e., objectively)” (2008: 1341). And Nichols and Folds-Bennett define objectivism as the view that “(i) true moral judgments are nonrelativistically true and (ii) some moral judgments are true” (2003: B24). “subjectivism” and “relativism” have both typically been meant to denote the position that is here referred to as “subjectivism”, i.e., the view that there are observer-dependent moral truths (Goodwin and Darley 2008: 1341; Nichols 2004: 7). In what follows I will hence use the label “realism” even when researchers themselves have spoken of “objectivism”, and I will use the label “subjectivism” when researchers themselves have spoken of “relativism”.

4 Studies involving subjects of different cultures include Beebe et al. (2015) and Sarkissian et al. (2011). By investigated subjects between the ages of 12 and 88 Beebe and Sackris (2016) attempted to shed light on the question of how intuitions about the existence of objective moral truths vary across the lifespan. Moreover, some studies have involved participants from Amazon Mechanical Turk, who vary in age too (e.g., Wright 2018).

5 Some researchers have successfully increased the variety of their item statements in some respects. Goodwin and Darley (2012), for example, included several statements about positive moral actions (in addition to the more usual statements about moral transgressions).

6 Some contemporary non-cognitivists argue that moral sentences are truth-apt in a non-robust (minimalist or deflationist) sense (e.g., Blackburn 2000: 79; Gibbard 2003: x). On such theories of moral truth, in saying of moral sentences that they are true we do not attribute any property to these sentences (such as the property of correctly representing the facts), but, for example, only reaffirm them.

7 Error theory has often been restricted to particular kinds of moral sentences (such as positive ones) (e.g., Joyce 2007a; Pigden 2007: 451), and has sometimes been stated in terms of these sentences being “untrue” rather than false (Joyce 2001: 6–9, 2007a). For a plausible defense of my above formulation see Loeb 2007.

8 In other research Beebe (2015) acknowledges this problem himself.

9 It may seem implausible that a significant proportion of subjects with non-cognitivist intuitions chose Beebe and Sackris’ realist answer. Isn’t the answer that it is possible for both parties of the disagreement to be correct much closer to their intuitions? Note, however, that even this anti-realist answer in an important sense contradicts non-cognitivism: it presupposes the existence of moral correctness. In the end the question of whether persons with non-cognitivist intuitions are more likely to respond that it is possible for both parties to be correct or that at least one of them must be mistaken is to be answered by empirical inquiry.

10 Conversely, moral facts are taken to be objective if and only if whether they obtain is *not* dependent on the mental states of observers. Suppose it were always wrong to break promises. Would breaking promises still be always wrong if I myself believed that it is not always wrong to break promises, if the culture in which I live dominantly believed that it is not always wrong to break promises, if humans did not respond with disapproval to breaking promises, and so on? If moral facts were objective these questions would have to be answered affirmatively.

11 Both individual subjectivism and cultural relativism can also be advocated in “agent” (as opposed to the above “appraiser”) variants. In these variants the truth of moral judgements is determined by the moral beliefs of the individual about who the judgement is made/by the moral beliefs of this individual’s culture (Gowans 2008). Harman’s version of cultural relativism is not fully appraiser-oriented, but involves elements of agent relativism as well.

12 Note that depending on one’s conception of objectivity, response dependence theory may also be regarded as a variant of realism. For discussion see Joyce 2007a.

13 Children between five and nine may not be able to grasp metaethical distinctions such as between realism and non-individualistic subjectivism. But note that even if true, this claim would not support the validity of Wainryb et al.’s measure. It would rather show that (young) children are not among the right kind of subjects for research on folk metaethics.

14 One may object that prior to psychological research on folk moral realism we lack any basis for discriminating between more and less prevalent variants of subjectivism. However, I believe that first, reasonable guesses can be made, and second, these guesses may always be revised in light of new evidence. If studies show, for example, that cultural relativism is only favored by a negligible proportion of people, and testing for cultural relativism is methodologically costly, subsequent research may justifiably disregard this variant of subjectivism.

15 Goodwin and Darley’s methodology has subsequently been adopted by several other researchers in the field, most importantly by Wright et al. (2013, 2014). They themselves developed and applied a new and in certain respects improved measure in Goodwin and Darley 2012.

16 Even this re-interpretation may be doubted. Sometimes the term “opinion” is intended to denote a truth-apt sentence (such as in “In my opinion Manchester United will win the league”) or a sentence that fails to be supported by good reasons (such as in “This is merely your opinion”) (Beebe 2015: 13–14). In case a subject understood “opinion” in one of these senses his/her “opinion or attitude” responses would (also) reflect cognitivist/epistemic intuitions rather than (purely) non-cognitivist ones (see also G6).

17 In addition, Nichols and Folds-Bennett (2003) also attempted to measure how much authority-independence subjects ascribed to moral vis-à-vis paradigmatic response-dependence sentences. For problems with this second measure of intuitions about moral response-dependence theory see Pölzler 2017: 469-471.

18 Another problem with Nichols and Folds-Bennett’s measure is that the above question may not have only prompted intuitions about the universalizability of moral sentences in the first place. It may have also directly addressed certain variants of anti-realism. For example, children may have responded that it was not good for one monkey to help another hurt monkey before there were people because they believed that goodness is determined by the moral beliefs of individuals (individual subjectivism) or by the moral beliefs of cultures (cultural relativism) — and “before there were people” there were no such moral beliefs.

19 Examples of reactive attitudes include approval, indignation and resentment.

20 To be clear, I do not advocate against measuring ordinary people’s intuitions about the moral universalism/relativism debate *per se*. Research of this kind is both psychologically and philosophically valuable. All that I am claiming is that investigating these intuitions does not improve our understanding of folk moral realism and anti-realism.

21 The distinctness of the moral realism/anti-realism and universalism/relativism debate has been explicitly noted and accounted for by Goodwin and Darley (2010: 162–165).

22 Some philosophers have argued that metaethical claims such as moral realism and anti-realism are actually only first-order moral claims in disguise (e.g., Dworkin 1996). Here I will proceed under the assumption that this view is false. First, there are good reasons to believe in moral realism and anti-realism’s moral neutrality (e.g., Bloomfield 2009; Shafer-Landau 2010). Second, this neutrality is predominantly accepted by philosophers as well as by psychologists (e.g., Goodwin and Darley 2012; Huemer 2005). Third, addressing this matter would lead us too far astray. And fourth, if it were true that moral realism and anti-realism are or entail judgements about what is morally right, wrong, good, bad, etc. then maybe a fundamentally different approach to measuring the folk’s intuitions about these positions would be in order (different from the approach that I attempt to improve in this paper).

23 This possibility is consistent with the finding that the supposedly metaethical ratings of many studies correlated with the strength of subjects’ first-order moral judgements. In particular, the stronger a subject agreed to a moral sentence the more likely s/he responded as a realist (or what the researchers interpreted as realism) to this sentence (Beebe et al. 2015: 397; Beebe and Sackris 2016: 21; Goodwin and Darley 2008: 1350, 1354; but see Goodwin and Darley 2010: 180; Wright et al. 2013).

24 In particular, it may be argued that even if subjects are successfully led to think on a metaethical level such intuitions arise automatically when one considers any moral sentence.

25 Stating answer options in terms of the disagreeing parties being right or wrong reduces first-order moral influences by avoiding explicitly moral concepts (which are more likely to trigger such intuitions). Disjunctive formulations signal that for the task at issue it does not matter which of the disagreeing parties is right. This is because disjunctions do not affirm any of the propositions that they connect. For example, “Either John is right and Fred is wrong, or Fred is right and John is wrong” leaves undecided whether John or Fred is right.

26 For example, both non-cognitivism and error theory entail that we cannot have moral knowledge, at least not in the ordinary sense of the term.

27 In her recent study on folk moral cognitivism/non-cognitivism (see below) Wright explains the non-epistemic nature of her questions as follows: “Consider […] the statement that ‘The earth is the only planet in our galaxy with life on it’. We simply don’t know at this point (and, indeed, we may never know) whether this statement accurately reflects a matter of fact (that is – whether it accurately reflects how many planets in our galaxy actually currently support life). So, we have no way of establishing whether the statement it is true or false – but, nonetheless, it is still truth-apt. It is either true or false – i.e., either the earth is the only planet in our galaxy with life on it or it isn’t.” (2018: 11). This explanation is well-formulated and likely succeeded in restricting epistemic influences on subjects’ responses.

28 In contrast to most other researchers who have addressed the cognitivism/non-cognitivism distinction, Wright did so intentionally and not as part of a broader measure of moral realism/anti-realism.

29 Consistent with the above worry, Wright’s results show a higher proportion of non-cognitivists than a previous study that addressed intuitions about the truth-aptness of moral sentences (Goodwin and Darley 2008).

30 Recall also Nichols and Folds-Bennett’s (2003) study on the prevalence of moral response-dependence theory which involved “yummy” — which is similar to “delicious” — as a paradigmatically response-dependent predicate (see G4).

31 In her above study on folk moral cognitivism/non-cognitivism Wright included a validity check. After subjects had read her instructions, she asked them to rate ten non-moral sentences as truth-apt or not truth-apt. Only subjects who rated at least nine sentences correctly were invited to participate in the main part of the study (2018: 12). While such an attempt to increase a study’s validity is to be welcomed in general (see G10), Wright’s check did not provide information about the effect of her instructions on subjects’ ratings of the truth-aptness of *moral* sentences, and it also did not alleviate the above-mentioned problems, for most of the test-sentences that she had classified as not truth-apt were practically normative, not uncontroversially truth-apt and widely contested as well (2018: 31).

32 The response that there is a correct answer about whether a given moral sentence is true is not only entailed by realism, but by all variants of cognitivism. Subjectivists believe that the correct answer to the question of whether some moral sentence is true can be “yes” (if the sentence correctly represents the subjective facts) or “no” (if the sentence does not correctly represent these facts). Error theorists believe that the correct answer is always “no”, i.e., that the sentence is not true. Moreover, the response that there is no correct answer about whether a given moral sentence is true should only appeal to non-cognitivists, for only according to them it does not make any sense to ascribe truth or falsity to such sentences at all.

33 Another possible strategy involves formulating scenarios, questions and answer-choices directly in terms of moral sentences’ representation of moral facts. Instead of asking subjects whether there can be a correct answer as to whether the moral sentence at issue is true, for example, they may ask whether there can be a correct answer as to whether the sentence represents a moral fact. On closer consideration, however, such reformulations only defer the above problem. Moral realists and anti-realists disagree about whether moral sentences “represent moral facts” in a robust sense of this notion, according to which for a sentence to represent a moral fact means that it reflects the exemplification of moral rightness, wrongness, goodness, badness, etc. But it is again unclear whether subjects assume this sense too. For example, might they not interpret phrases such as “represents a moral fact” as expressing only the reaffirmation of a moral statement (deflationist theory of moral facts; see, e.g., Blackburn 2000: 79; Dworkin 1996: 87–98)? (Sinclair 2012: 168). There thus seems to be no way around testing the understanding of *some* of subjects’ philosophical concepts.

34 One result that might emerge from these investigations into people’s understanding of moral truth is that they are moral truth pluralists; for example, that they take some moral sentences to be true in virtue of their correspondence to moral facts (correspondence theory) and others in virtue of their simply being affirmed by a speaker (deflationist theory). This finding would complicate research on folk moral realism. It would mean that in order to conclusively determine subjects’ intuitions about moral realism researchers would have to test their understanding of moral truth for each of the item statements that they use in the study. Only responses to item statements that receive a correspondence-theoretic interpretation could then be taken to be indicative of either moral realism or anti-realism. Though feasible, such a survey design would certainly be extensive and complex. Researchers would have to weigh the resulting benefits in terms of construct validity against potential pragmatic costs.

35 In fact, the worry afflicts *any* psychological study on ordinary people’s philosophical intuitions (Bengson 2013).

36 Subjects have been found to be more likely to rate a moral statement as true and to rate one of the parties of a disagreement about this statement as wrong when they take this statement to be widely accepted (Goodwin and Darley 2012: 253–254; Wright et al. 2013: 349) and when they themselves strongly agree with the statement (Goodwin and Darley 2008: 1350, 1354).
